# Characterization of high healthcare utilizer groups using administrative data from an electronic medical record database

**DOI:** 10.1186/s12913-019-4239-2

**Published:** 2019-07-05

**Authors:** Sheryl Hui-Xian Ng, Nabilah Rahman, Ian Yi Han Ang, Srinath Sridharan, Sravan Ramachandran, Debby D. Wang, Chuen Seng Tan, Sue-Anne Toh, Xin Quan Tan

**Affiliations:** 1 Centre for Health Services and Policy Research, Saw Swee Hock School of Public Health, National University of Singapore and National University Health System, Singapore, Singapore; 2 Regional Health System Office, National University Health System, Singapore, Singapore; 3 Saw Swee Hock School of Public Health, National University of Singapore and National University Health System, Singapore, Singapore

**Keywords:** Healthcare, Utilization, Segmentation, Super-utilizers, Cost, Expenditure, Administrative, Persistence

## Abstract

**Background:**

High utilizers (HUs) are a small group of patients who impose a disproportionately high burden on the healthcare system due to their elevated resource use. Identification of persistent HUs is pertinent as interventions have not been effective due to regression to the mean in majority of patients. This study will use cost and utilization metrics to segment a hospital-based patient population into HU groups.

**Methods:**

The index visit for each adult patient to an Academic Medical Centre in Singapore during 2006 to 2012 was identified. Cost, length of stay (LOS) and number of specialist outpatient clinic (SOC) visits within 1 year following the index visit were extracted and aggregated. Patients were HUs if they exceeded the 90th percentile of any metric, and Non-HU otherwise. Seven different HU groups and a Non-HU group were constructed. The groups were described in terms of cost and utilization patterns, socio-demographic information, multi-morbidity scores and medical history. Logistic regression compared the groups’ persistence as a HU in any group into the subsequent year, adjusting for socio-demographic information and diagnosis history.

**Results:**

A total of 388,162 patients above the age of 21 were included in the study. Cost-LOS-SOC HUs had the highest multi-morbidity and persistence into the second year. Common conditions among Cost-LOS and Cost-LOS-SOC HUs were cardiovascular disease, acute cerebrovascular disease and pneumonia, while most LOS and LOS-SOC HUs were diagnosed with at least one mental health condition. Regression analyses revealed that HUs across all groups were more likely to persist compared to Non-HUs, with stronger relationships seen in groups with high SOC utilization. Similar trends remained after further adjustment.

**Conclusion:**

HUs of healthcare services are a diverse group and can be further segmented into different subgroups based on cost and utilization patterns. Segmentation by these metrics revealed differences in socio-demographic characteristics, disease profile and persistence. Most HUs did not persist in their high utilization, and high SOC users should be prioritized for further longitudinal analyses. Segmentation will enable policy makers to better identify the diverse needs of patients, detect gaps in current care and focus their efforts in delivering care relevant and tailored to each segment.

**Electronic supplementary material:**

The online version of this article (10.1186/s12913-019-4239-2) contains supplementary material, which is available to authorized users.

## Background

High healthcare utilizers are a small group of patients who impose a disproportionately high burden on the healthcare system due to their elevated resource use, and often have unmet care needs or receive unnecessary care [1]. To design policies to address these issues, high healthcare utilization and its drivers have been studied extensively in recent years. The definition of high utilization has been heterogeneous. The choice of metric used to measure utilization often differs and depends on the disease or health service context. As distributions of healthcare cost and utilization incurred by patients are often skewed [2–4], the approach of defining high utilizers (HUs) as patients in the top percentiles of healthcare cost has been commonly adopted. Most studies use cost to identify HUs as it can be regarded as a measure of utilization intensity [5, 6]. It also gives a direct economic perspective (e.g. potential cost savings and impact on government funding) to the problem at hand [7]. The percentile threshold for cost used to identify HUs varies between studies, ranging from the top 5% of patients [7–16] to the top 20% [17], with top 10% being the most common definition used [1, 7, 11, 15, 18–26]. Cost is a good measure of utilization and can serve as a proxy for utilization across different resource types (e.g. inpatient admissions, outpatient visits and procedures). However, as cost would be heavily influenced by the number of inpatient bed days incurred by a patient, looking at cost alone may not provide a complete picture of utilization volume. Other metrics commonly used to identify HUs include outpatient visits to clinics [27], emergency attendances [28–30], and inpatient utilization such as readmissions within a certain period [31–33] or length of stay (LOS) [34–36]. There are few papers that examine multiple metrics simultaneously [23, 37–39]. Examining other metrics of utilization in tandem with cost will allow policymakers and clinicians to look at multiple dimensions of resource use [37, 40], and understand the different underlying drivers to get a more comprehensive understanding of healthcare utilization. Furthermore, segmentation of a patient population using multiple metrics would create smaller groups of patients with largely similar utilization patterns and characteristics, facilitating targeting and tailoring of interventions for effective use of resources [41].

With the increasing adoption of electronic medical record (EMR) systems in hospitals [42–44], comprehensive administrative cost and utilization data over multiple years are now more readily available. Researchers and health systems can use this information to segment the general patient population and address the diverse needs of each patient segment [41]. Segmentation will help identify homogenous patient subpopulations and provide knowledge on their characteristics, needs and trajectories over time. This knowledge would then support development and implementation of interventions targeted at each subpopulation, such that the interventions are more tailored to individual needs, and likely to be of greater impact [41, 45–48]. In the long run, this would also facilitate program evaluation and outcomes tracking for each group [48].

While examining high utilization in a cross-sectional manner allows us to understand the profiles of HUs, observing HUs longitudinally would provide valuable information on how utilization per patient accumulates over time, how patients transit between HU groups and how patients’ utilizations change with their transitions. The definition of persistence of HU behavior differs widely between studies, with one definition being recurrence as a HU in the subsequent year [4, 9, 19, 49]. Identification of persistent users is pertinent as interventions on HUs have not been shown to be efficacious or cost-effective potentially due to regression to the mean in majority of patients [20, 50]. Hence, insights from these longitudinal analyses could potentially inform how healthcare systems can better design and target interventions for HUs.

This study will demonstrate the use of cost and utilization metrics to segment a patient population into groups of high healthcare utilizers, based on 1 year’s patterns of hospital-based resource use in an Academic Medical Center (AMC) in Singapore. The groups’ socio-demographic characteristics, utilization patterns and medical history will be described for comparison. The comparisons will illustrate the benefits of using multiple metrics to identify different HU profiles, highlight the healthcare needs associated with each profile and their subsequent longitudinal behaviors. This work provides multifaceted insights on the characteristics of high healthcare utilizers, which will inform program and policy development, and the identification of the correct subgroups for more targeted interventions.

## Methods

Data analyzed was from a hospital administrative database in an AMC in Singapore for the period of 2006 to 2013. Ethics approval was obtained from the review board of the healthcare cluster. Details of preparation and processing of the database are described elsewhere [51]. Patients aged 21 years and above and had at least one record of either an inpatient admission, specialist consultation, therapy visit or emergency attendance between 1st January 2006 and 31st December 2012 were included in the study. The index visit to the AMC for each patient, defined as the first visit occurring on or after 1st January 2006 and before 1st January 2013, was identified. All visits within 1 year following the index visit were then extracted. Multi-morbidity measures were included using the Charlson Comorbidity Index (CCMI) score adapted by Quan [52, 53], as well as a polypharmacy score (PPS) measuring the highest number of unique dispensed medications a patient was ever prescribed in a visit [54]. Diagnosis codes were aggregated into Clinical Classification Software (CCS) groups for ease of reporting [55].

Metrics of hospital utilization described in this study were the number of inpatient admissions, LOS in inpatient care, Specialist Outpatient Clinic (SOC) visits, Emergency Department (ED) attendances, as well as healthcare costs accumulated over all hospital settings incurred per patient. Healthcare costs incurred by the patient were proxied by the bill charged to the patient before any Government subsidies or third-party payments (e.g. payments by insurers or employers) and were presented in Singapore dollars (S$), adjusted to 2015 figures. LOS per inpatient admission was computed as the total duration of hospitalization that was not attributed to short stays (e.g. day surgery and endoscopy). This was in line with methodology adopted by the Organization for Economic Co-operation and Development (OECD) where short-term treatments were excluded due to variation in clinical environments for these treatments across countries [56]. SOC visits were defined as outpatient specialist consultations within the AMC. Primary care information was not available at time of the study. For analysis, cost and utilization from all visits within the observed year were aggregated and reported for each patient.

### Classification into HU groups

With no universal set of metrics to measure high resource use, Cost, LOS and number of SOC visits were selected as the metrics for identification of HUs to capture multiple facets of hospital utilization, and the threshold for high utilization was set at 90th percentile of each metric as it is the most common threshold used in literature. Cost would provide a measure of the global economic burden on the hospital system, including all inpatient-related resources, all ambulatory services including emergency department visits and outpatient visits, manpower, consumables, pharmaceuticals and procedures. To measure multiple aspects of utilization in addition to cost, LOS and SOC visits incurred would also be included, as they measure resource and service use specifically in the inpatient and outpatient settings respectively. Furthermore, we chose to use LOS as a metric of inpatient utilization instead of the number of admissions as LOS would account for the variation in number of patient days per admission, which would more accurately reflect the intensity of inpatient resource use in comparison to using only number of admissions as a metric [57]. We chose not to include number of ED attendances as a specific metric for identification of HUs. As frequent ED users, commonly defined as patients who incur 4 or 5 visits in literature, constitute only 5% of a patient cohort, the 90th percentile cut off would capture a substantially larger subgroup and not distinguish the high users sufficiently well [58–64]. Only patients with admissions were included in computation of the 90th percentile of LOS. Patients who exceeded the threshold for any of the three metrics were classified as HUs for the observed year. Patients who were HUs in all three metrics were labelled as Cost-LOS-SOC HUs, while patients who satisfied the criteria in only two metrics were Cost-LOS, Cost-SOC or LOS-SOC HUs correspondingly. Patients who satisfied the HU criteria in only a single metric were classified as Cost, LOS or SOC HUs accordingly. This ensured membership in each HU group was mutually exclusive. Patients who incurred cost or utilization below the 90th percentile for all metrics were classified as the Non-HU group.

The patient cohort, partitioned by their HU group memberships, were described in terms of their cost and utilization patterns, and profiled using their socio-demographic information, multi-morbidity scores and medical history. Age at patients’ first visit in the system was reported. As patients seeking care in public healthcare institutions in Singapore may receive government subsidies with the quantum dependent on their household income [65], we classified patients into three levels of subsidy based on the amount of subsidised treatment they received. Patients would have received either only subsidised treatment, only unsubsidised treatment, or a mix of both subsidised and unsubsidised treatment. Socio-economic status (SES) of patients was described by their housing type, derived from their last known postal code information in the system. Housing in Singapore can be tiered into private housing, public housing and public rental housing. Private housing caters mainly to the upper-middle to upper income groups. Public housing caters to the middle-class population and public rental housing to the low-income groups. Each residential property or block in Singapore is uniquely assigned a postal code akin to an address, and neither public housing nor public rental housing share postal codes with private housing types. Approximately 80% of residents in Singapore live in public housing, with the smallest housing type being 1-room rental flats and the largest being executive flats with three bedrooms [66]. As the cost of these flats increase with size, housing type can serve as a proxy of SES. In this study, we identified the housing type present at each postal code using a map of postal codes and their respective housing types validated from a separate study, and for public housing blocks with more than one flat type, the housing type assigned was the flat type with the largest proportion in the block [51]. We categorized housing type in increasing order of SES: 1/2-room flats, 3-room flats and larger, and private housing. Hence, given a patient’s postal code, we were able to determine their corresponding housing type as a proxy of SES. Multi-morbidity measures, cost and utilization were reported in terms of median and interquartile range (IQR) specified as a range spanning the first quartile to the third quartile. The proportion of patients who died within the observed year was also reported. To describe the medical history of each group, the primary diagnosis for each visit (i.e. medical condition the patient sought healthcare service for) was extracted for each patient. We looked at common diagnoses within each diagnosis groups, in terms of both the number of patients who had ever sought care for that condition, as well as separately in terms of the number of visits attributed to that condition. Within each HU group, the five most common conditions recorded were reported.

### Persistence of HU behavior

After describing the demographic and clinical profiles of the HU groups, we were interested in whether the HU groups had differing extents of persistence in the subsequent year. Persistence was defined as membership in any HU group in a subsequent observed year. All cost and utilization incurred during a one-year period following the first observed year was aggregated for each patient, and patients were classified as HUs if they exceeded the same HU thresholds from the first observed year. The proportion of persistent patients in each HU group were reported. To compare the tendencies for persistence between the HU groups, logistic regression models were built. Patients who died within the first observed year or had missing socio-demographic information were excluded from the analysis, and patients with no utilization in the subsequent year were subsumed under the Non-HU status. Missing CCMI information was assumed to be 0. First, a model (Model 0) was constructed to look at the associations between each first observed year HU group and the outcome of subsequent year HU status. Model 1 was estimated by adjusting Model 0 for the socio-demographic characteristics and multi-morbidity measures and removing factors which were not statistically significant at 0.1% significance level (*p* < 0.001) to obtain a parsimonious model. Model 2 was then built by further adjusting Model 1 for common HU conditions, and similarly factors which were no longer significant at 0.1% significance level (*p* < 0.001) were removed. Odds ratios (ORs) for each factor and the corresponding 99% confidence intervals (CIs) were reported. McFadden’s pseudo-R^2^ for each model was reported [67], and likelihood ratio tests (LRTs) were conducted for comparison of model fit between Model 0 and 1, as well as Model 1 and 2 [68]. All statistical analyses were carried out using RStudio using the *dplyr* package [69, 70].

## Results

A total of 388,162 patients above the age of 21 recorded at least one visit to the hospital over the study period. The patient population was divided into eight distinct segments, with seven groups of HUs and one group of Non-HUs. The utilization patterns of patients according to the HU grouping are described in Table 1. Non-HUs constitute 83% of all patients and 25% of all costs during the first observed year. Few patients had inpatient utilization, and outpatient utilization was an average of 1 SOC visit or ED attendance. Cost HUs accounted for almost 16% of total costs despite constituting less than 4% of the cohort. The median costs for these HUs was S$16,591 and median inpatient utilization was 1 inpatient admission and 7 days of LOS. Similarly, most LOS HUs only had 1 inpatient admission, but their median bill was lower at S$9073 and LOS was longer at 19 days. LOS-SOC HUs incurred similar bill sizes and inpatient utilization as LOS HUs, but had additional high SOC usage (LOS HU: median: 1 visit; LOS-SOC HU: 9 visits). SOC HUs, due to the large group size (6.7%), accounted for 8% of all cost despite having zero inpatient utilization and ED attendances on average. Cost-SOC HUs generally incurred more utilization in comparison to SOC HUs (median inpatient admissions: 1; LOS: 6 days; SOC visits: 12). Cost-LOS HUs incurred more cost and inpatient utilization than Cost HUs, at a median cost of S$31,762, and median inpatient utilization of 2 admissions and 27 days of LOS. The Cost-LOS-SOC patients incurred the highest utilization across all metrics (median cost: S$49,248; inpatient admissions: 3; LOS: 29; SOC visits: 13; ED attendances: 2).

**Table 1:** Cost and utilization patterns of high utilizer (HU) groups

	Total (*N* = 388,162)	Non-HU (*N* = 322,905)	Cost (*N* = 14,647)	LOS (*N* = 217)	SOC (*N* = 26,179)	LOS-SOC (*N* = 44)	Cost-LOS (*N* = 6008)	Cost-SOC (*N* = 12,892)	Cost-LOS-SOC (*N* = 5270)
Population size	100%	83.2%	3.8%	0.1%	6.7%	0.1%	1.5%	3.3%	1.4%
% of total cost incurred	100%	25.1%	15.8%	0.1%	7.8%	0.0%	15.6%	16.9%	18.6%
Gross amount (S$), median (IQR)	824	525	16,591	9073	5234	8991	31,762	19,263	49,248
	(281-3344)	(256-1642)	(13,380-22,119)	(7901-10,013)	(2956-7554)	(7856-9982)	(20,876-52,854)	(14,015-28,203)	(33,145-74,628)
Inpatient admissions, median (IQR)	0 (0–1)	0 (0–0)	1 (1–1)	1 (1–2)	0 (0–1)	1 (1–2)	2 (1–3)	1 (1–2)	3 (2–4)
LOS, median (IQR)	0 (0–1)	0 (0–0)	7 (4–11)	19 (17–22)	0 (0–3)	20 (18–21)	27 (20–40)	6 (2–10)	29 (21–44)
SOC visits, median (IQR)	1 (0–3)	1 (0–2)	3 (1–5)	1 (0–3)	9 (8–12)	9 (8–10)	2 (0–4)	12 (8–18)	13 (9–19)
ED attendances, median (IQR)	1 (0–1)	1 (1–1)	1 (0–1)	1 (1–2)	0 (0–1)	1 (1–2)	1 (1–3)	0 (0–1)	2 (1–3)

The socio-demographic profiles of the HU groups are presented in Table 2. Overall, most Non-HU patients were aged below 40 and had low multi-morbidity (median age: 36; median CCMI: 0; median PPS: 2). The majority were male, Chinese, sought at least some subsidised services or stayed in 3-room public housing or larger (male: 59.6%; Chinese: 58.0%; only unsubsidised treatment: 11.7%; 3-room and larger: 62.6%). Death within the observed year and persistence was low (death: 1.4%; persistence: 2.2%). Cost HUs in comparison were older, had a larger proportion of patients who sought only unsubsidised treatment and a tenth of the patients died during the year (median age: 55; only unsubsidised treatment: 19.7%). LOS HUs were also older than Non-HUs, mostly female, and a larger proportion were Chinese (median age: 55; male: 39.2%; Chinese: 72.8%). Almost all sought at least some subsidised services (99.5%), and 9.2% of patients died during the year. LOS-SOC HUs were similar to LOS HUs in terms of race, ethnicity and SES, but were younger with a median age of 32, and all patients survived. SOC HUs were similar in demographic profile to Non-HUs, but had more female patients and the highest proportion of patients who sought only unsubsidised treatment (male: 37.3%; unsubsidised treatment: 31.0%). Persistence was also prevalent in 15% in SOC HUs, which was substantially higher than in the Non-HUs. Cost-SOC patients were older than SOC HUs, had more males and exhibited more persistence in comparison (median age: 51; male: 52.7%; persistence: 25.2%). Cost-LOS patients were the oldest, had the highest multi-morbidity and the highest proportion of patients living in 1/2-room flats (median age: 66; median CCMI: 2; median PPS: 25; 1/2-room flat: 6.2%). They also had the highest death rate among all groups within the first year (27.1%). Cost-LOS-SOC HUs had the highest multi-morbidity, and a third of patients persisted as HUs into the second year (median CCMI: 2; median PPS: 29; persistence: 35.4%).

**Table 2:** Characteristics of Year 1 high utilizer (HU) groups

	Total (*N* = 388,162)	Non-HU (*N* = 322,905)	Cost (*N* = 14,647)	LOS (*N* = 217)	SOC (*N* = 26,179)	LOS-SOC (*N* = 44)	Cost-LOS (*N* = 6008)	Cost-SOC (*N* = 12,892)	Cost-LOS-SOC (*N* = 5270)
Age at first visit	38 (27–53)	36 (27–50)	55 (44–67)	55 (33–76)	36 (28–53)	32 (27–42)	66 (51–77)	51 (37–63)	58 (47–69)
PPS^a^, median (IQR)	3 (1–6)	2 (0–4)	15 (10–22)	9 (6–13)	7 (3–11)	6 (4–8)	25 (17–40)	14 (9–20)	29 (20–41)
	*N = 354,569*	*N = 292,096*	*N = 14,622*		*N = 23,469*			*N = 12,843*	
CCMI^b^, median (IQR)	0 (0–0)	0 (0–0)	1 (0–2)	0 (0–1)	0 (0–0)	0 (0–0)	2 (1–4)	1 (0–2)	2 (1–5)
Gender									
Female	162,057 (41.7%)	130,395 (40.4%)	4615 (31.5%)	132 (60.8%)	16,404 (62.7%)	20 (45.5%)	2415 (40.2%)	6104 (47.3%)	1972 (37.4%)
Male	226,105 (58.3%)	192,510 (59.6%)	10,032 (68.5%)	85 (39.2%)	9775 (37.3%)	24 (54.5%)	3593 (59.8%)	6788 (52.7%)	3298 (62.6%)
Race									
Chinese	226,849 (58.4%)	187,417 (58.0%)	8548 (58.4%)	158 (72.8%)	15,376 (58.7%)	29 (65.9%)	3750 (62.4%)	8110 (62.9%)	3461 (65.7%)
Indian	50,475 (13.0%)	42,748 (13.2%)	1482 (10.1%)	21 (9.7%)	4131 (15.8%)	4 (9.1%)	513 (8.5%)	1172 (9.1%)	404 (7.7%)
Malay	45,709 (11.8%)	38,062 (11.8%)	1926 (13.1%)	15 (6.9%)	2806 (10.7%)	5 (11.4%)	933 (15.5%)	1270 (9.9%)	692 (13.1%)
Others	65,129 (16.8%)	54,678 (16.9%)	2691 (18.4%)	23 (10.6%)	3866 (14.8%)	6 (13.6%)	812 (13.5%)	2340 (18.2%)	713 (13.5%)
Treatment type									
Subsidised only	263,111 (67.8%)	231,507 (71.7%)	8180 (55.8%)	167 (77.0%)	10,754 (41.1%)	23 (52.3%)	4403 (73.3%)	4987 (38.7%)	3090 (58.6%)
Unsubsidised only	53,331 (13.7%)	37,901 (11.7%)	2885 (19.7%)	1 (0.5%)	8110 (31.0%)	2 (4.5%)	439 (7.3%)	3598 (27.9%)	395 (7.5%)
Both subsidised and unsubsidised	71,720 (18.5%)	53,497 (16.6%)	3582 (24.5%)	49 (22.6%)	7315 (27.9%)	19 (43.2%)	1166 (19.4%)	4307 (33.4%)	1785 (33.9%)
Housing type									
1/2-room flats	10,106 (2.6%)	8087 (2.5%)	577 (3.9%)	7 (3.2%)	550 (2.1%)	0 (0%)	375 (6.2%)	289 (2.2%)	221 (4.2%)
3-room flats and larger	245,741 (63.3%)	202,252 (62.6%)	9093 (62.1%)	163 (75.1%)	18,680 (71.4%)	32 (72.7%)	3956 (65.8%)	7872 (61.1%)	3693 (70.1%)
Private	45,714 (11.8%)	37,736 (11.7%)	1414 (9.7%)	14 (6.5%)	3838 (14.7%)	3 (6.8%)	427 (7.1%)	1857 (14.4%)	425 (8.1%)
Unknown	86,601 (22.3%)	74,830 (23.2%)	3563 (24.3%)	33 (15.2%)	3111 (11.9%)	9 (20.5%)	1250 (20.8%)	2874 (22.3%)	931 (17.7%)
Death	8722 (47.5%)	4625 (1.4%)	1460 (10.0%)	20 (9.2%)	90 (0.3%)	0 (0%)	1626 (27.1%)	247 (1.9%)	654 (12.4%)
Persistence of HU	16,052 (4.1%)	7108 (2.2%)	774 (5.3%)	19 (8.8%)	3819 (14.6%)	11 (25.0%)	651 (10.8%)	3244 (25.2%)	1863 (35.4%)

^a^*PPS* Polypharmacy score, ^b^*CCMI* Charlson Comorbidity Index score

Table 3 illustrates the five most common conditions patients in each HU group that were ever diagnosed in the first year. External injuries were common primary diagnoses in the Non-HU group. Cardiovascular disease was prevalent among the Cost HUs (Coronary atherosclerosis: 20.1%; Acute myocardial infarction: 19.6%). SOC HUs were commonly diagnosed with routine ambulatory conditions, with predominantly pregnancy related conditions (Normal pregnancy: 13.3%; complications: 4.4%), while Cost-SOC HUs were commonly diagnosed with complex ambulatory conditions such as cancer and female infertility (Cancer of breast: 7.4%; Female infertility: 5.0%). Most LOS and LOS-SOC HUs were diagnosed with at least one mental health condition, with mood disorders highly prevalent (LOS: 24.0%; LOS-SOC: 52.3%). For Cost-LOS and Cost-LOS-SOC HUs, common conditions were cardiovascular disease, acute cerebrovascular disease as well as pneumonia (Cardiovascular: Cost-LOS: 8.2%, Cost-LOS-SOC: 10.9%; Cerebrovascular: Cost-LOS: 17.5%; Cost-LOS-SOC: 8.3%; Pneumonia: Cost-LOS: 13.9%; Cost-LOS-SOC: 7.9%). Common diagnoses ranked by visit frequency revealed similar trends. The only exception was the prevalent conditions in Cost-LOS-SOC HUs were lymphoma, leukemia, colon cancer and renal failure (Additional file 1).

**Table 3:** Top 5 common conditions in Year 1 high utilizer (HU) groups

HU Group	Condition	Patients ever diagnosed (%)
Non-HU	Superficial injury; contusion	27,049 (8.4%)
	Other upper respiratory infections	14,778 (4.6%)
	Sprains and strains	14,546 (4.5%)
	Non-infectious gastroenteritis	12,371 (3.8%)
	Open wounds of extremities	11,447 (3.5%)
Cost	Coronary atherosclerosis and other heart disease	2946 (20.1%)
	Acute myocardial infarction	2877 (19.6%)
	Acute cerebrovascular disease	879 (6.0%)
	Essential hypertension	781 (5.3%)
	Fracture of lower limb	604 (4.1%)
LOS	Mood disorders	52 (24.0%)
	Schizophrenia and other psychotic disorders	36 (16.6%)
	Residual codes; unclassified	17 (7.8%)
	Pneumonia^a^	13 (6.0%)
	Intracranial injury	12 (5.5%)
SOC	Normal pregnancy and delivery	3490 (13.3%)
	Cataract	1379 (5.3%)
	Other complications of birth	1151 (4.4%)
	Fracture of upper limb	1055 (4.0%)
	Gastritis and duodenitis	743 (2.8%)
LOS-SOC	Mood disorders	23 (52.3%)
	Schizophrenia and other psychotic disorders	14 (31.8%)
	Other eye disorders	5 (11.4%)
	Superficial injury; contusion	5 (11.4%)
	Burns	4 (9.1%)
Cost-LOS	Acute cerebrovascular disease	1049 (17.5%)
	Pneumonia^a^	838 (13.9%)
	Urinary tract infections	546 (9.1%)
	Coronary atherosclerosis and other heart disease	493 (8.2%)
	Acute myocardial infarction	417 (6.9%)
Cost-SOC	Cancer of breast	955 (7.4%)
	Coronary atherosclerosis and other heart disease	925 (7.2%)
	Female infertility	650 (5.0%)
	Fracture of upper limb	619 (4.8%)
	Essential hypertension	581 (4.5%)
Cost-LOS-SOC	Coronary atherosclerosis and other heart disease	572 (10.9%)
	Acute cerebrovascular disease	440 (8.3%)
	Pneumonia^a^	414 (7.9%)
	Essential hypertension	398 (7.6%)
	Septicemia (except in labour)	363 (6.9%)

^a^except that caused by tuberculosis or sexually transmitted disease

We then sought to identify factors associated with persisting as a HU into the subsequent year. Generally, persistent and non-persistent HUs differed in socio-demographic characteristics and prevalence of common HU conditions (Additional file 2). Of all patients, 16,052 (4.2%) patients were HU in a subsequent year. From Table 4, Model 0 revealed that HUs across all groups were more likely to persist as a HU in any group in the subsequent year, compared to Non-HUs. The weakest association was seen in the Cost HUs, while the strongest association was seen in the Cost-LOS-SOC HUs (Cost: OR = 2.73, 99% CI: 2.45–3.03; Cost-LOS-SOC: 31.59, 99% CI: 29.02–34.37), with generally stronger associations seen in groups with high SOC utilization (LOS-SOC: 16.72, 99% CI: 6.26–38.93; Cost-SOC: 16.30, 99% CI: 15.31–17.35). After adjusting Model 0 for all socio-demographic factors and multi-morbidity scores, housing type was removed from the model due to lack of statistical significance. The resulting model, Model 1, revealed that the trends in persistence among the HU groups had remained but decreased in strength across all groups except for LOS-SOC HUs (Model 0: OR: 16.72, 99% CI: 6.26–38.93; Model 1: 17.28, 99% CI: 6.39–40.94). The LRT revealed that Model 1 exhibited significantly better fit than Model 0 (*p* < 0.001). Adjusting Model 1 for the 26 common HU conditions and further refining the model to retain only statistically significant factors, only 13 conditions remained in Model 2. The same trend in varying tendencies in persistence among the HU groups was observed. A diagnosis of breast cancer, mood disorders, hypertension or female infertility was also associated with a higher likelihood of persistence (Cancer of breast: 1.28, 99% CI: 1.09–1.51; mood disorders: 1.45, 99% CI: 1.11–1.85; hypertension: 1.53, 99% CI: 1.36–1.71; female infertility: 1.72, 99% CI: 1.39–2.12). The LRT revealed that Model 2 exhibited significantly better fit than Model 1 (*p* < 0.001).

**Table 4:** Factors associated with persistence as a HU into a subsequent year

	Odds ratio (99% confidence interval)		
	Model 0	Model 1^c^	Model 2^c^
Non-HU	1.00	1.00	1.00
Cost	2.73 (2.45–3.03)	1.85 (1.64–2.07)	1.77 (1.57–1.98)
LOS	5.67 (2.92–9.97)	3.60 (1.83–6.44)	3.36 (1.71–6.02)
SOC	8.20 (7.75–8.68)	6.71 (6.31–7.12)	7.40 (6.95–7.87)
LOS-SOC	16.72 (6.26–38.93)	17.28 (6.39–40.94)	14.04 (5.07–34.39)
Cost-LOS	7.41 (6.55–8.35)	4.07 (3.51–4.71)	3.97 (3.42–4.61)
Cost-SOC	16.30 (15.31–17.35)	9.83 (9.08–10.63)	9.43 (8.68–10.25)
Cost-LOS-SOC	31.59 (29.02–34.37)	17.61 (15.50–20.01)	16.82 (14.78–19.15)
Age at first visit		1.01 (1.01–1.02)	1.01 (1.01–1.01)
CCMI^b^		1.27 (1.25–1.29)	1.24 (1.22–1.26)
PPS^a^		0.99 (0.99–0.99)	0.99 (0.99–1.00)
Gender			
Female		1.00	1.00
Male		0.71 (0.68–0.75)	0.72 (0.69–0.75)
Race			
Chinese		1.00	1.00
Indian		1.23 (1.14–1.33)	1.26 (1.17–1.36)
Malay		1.02 (0.95–1.09)	1.05 (0.97–1.12)
Others		0.93 (0.86–1.01)	0.93 (0.86–1.00)
Nationality			
Foreigner		1.00	1.00
Singaporean		2.35 (2.21–2.51)	2.27 (2.12–2.42)
Treatment type			
Subsidised only		1.00	1.00
Both subsidised and unsubsidised		1.42 (1.34–1.51)	1.46 (1.37–1.55)
Unsubsidised only		1.89 (1.78–2.01)	1.85 (1.74–1.97)
Common HU conditions			
Other puerperium complications of birth			0.35 (0.26–0.47)
Normal pregnancy and delivery			0.41 (0.34–0.49)
Open wounds of extremities			0.48 (0.38–0.59)
Fracture of upper limb			0.48 (0.39–0.58)
Burns			0.51 (0.28–0.84)
Fracture of lower limb			0.66 (0.54–0.80)
Other eye disorders			0.70 (0.56–0.87)
Cataract			0.70 (0.59–0.82)
Superficial injury contusion			0.70 (0.61–0.79)
Cancer of breast			1.28 (1.09–1.51)
Mood disorders			1.45 (1.11–1.85)
Essential hypertension			1.53 (1.36–1.71)
Female infertility			1.72 (1.39–2.12)
Model comparison indices			
McFadden’s R^2^	0.16	0.20	0.21
Residual deviance	111,630	106,351	105,539
Residual degrees of freedom	379,427	379,417	379,404
Likelihood ratio test (χ^2^)	–	< 0.001	< 0.001

^a^*PPS* Polypharmacy score, ^b^*CCMI* Charlson Comorbidity Index score, ^c^Model 1: Adjusted for socio-demographics and multi-morbidity; Model 2: Adjusted for socio-demographics, multi-morbidity and common HU conditions

## Discussion

HUs of healthcare services are a diverse group and can be further segmented into different subgroups based on utilization metrics available in most hospital administrative databases, such as cumulative cost, length of stay or outpatient visits. Segmentation by these utilization metrics revealed differences in socio-demographic characteristics, varying persistence in high utilization and distinct variations in disease profiles of patients. Our results showed that the HU groups exhibit differences in age and comorbidity. High-cost groups were generally older and of higher multi-morbidity in comparison to the low-cost groups, which is consistent with other studies associating older age and multi-morbidity with higher healthcare costs [1, 3, 71–73]. As few studies define high utilization using multiple metrics simultaneously [37, 40], our study adds meaningful insights into the characteristics of patients in different HU groups, such as the variation in extent of multi-morbidity between the groups. However, while socio-demographic factors have been shown to be associated with HUs in other populations [9, 14, 17, 74], this was not as apparent in our population as housing type, as a proxy of SES, does not appear to be a differentiating factor across the groups.

Our results also revealed that the HU groups have different disease profiles. The disease profile of the Cost-LOS and Cost-LOS-SOC HUs, together with the older average age, higher CCMI and substantial death rate, suggest a frail elderly archetype similar to clusters with advanced age and high prevalence of complex chronic conditions found in recent segmentation studies [39, 75, 76]. The finding of acute cardiac events as a one-off high-cost condition was consistent with other studies. Similarly, common resource-intensive conditions such as cancers and renal failure were observed to be among the most prevalent conditions in the Cost-SOC and Cost-LOS-SOC HUs when the number of visits per condition was taken into account [13, 19, 26]. The LOS and LOS-SOC HUs were found to be primarily patients with diagnosed mental illness. The underlying drivers of high utilization and required interventions for patients admitted to the psychiatric wards and patients admitted to the general wards would differ. For patients admitted for psychiatric conditions, interventions such as rapid psychiatric review upon admission could potentially reduce inpatient stay in the psychiatric wards [77, 78]. For the patients admitted for non-psychiatric diagnoses, the long stay may be driven by factors such as poor access to appropriate psychosocial care [37], suggesting that instead of cost reduction measures, patients may instead benefit from an integrated model of care to reduce the burden on acute inpatient care [79, 80].

A key finding is that most HUs in our patient cohort do not persist in their high utilization, precluding intervention after identification in the first observed year where they incur substantial resource use. This finding is consistent with other studies that have shown that even within HUs, there exists a small group of high-risk persistent users incurring a disproportionate amount of cost [7, 20, 73, 81, 82]. HUs were more likely to recur as a HU in any group in the subsequent year, and this phenomenon was more pronounced in groups with high SOC utilization in the first observed year. Our multivariable model suggested that patients incurring high resource usage in the SOC setting, such as for treatment of female infertility, should be prioritized for further longitudinal analyses to better understand their utilization trajectories, with the aim of developing programs with these specific characteristics in mind. In addition, current disease management processes for hypertension and mood disorders should also be flagged for further analyses and refinement to address the tendency for persistence in these patients. Future studies would also seek to examine persistence of HU behavior over a longer duration and the different trajectories of each subgroup to inform intervention design and targeting.

As utilization patterns may be driven by patients’ disease type, progression and management [73], interventions to reduce excess resource use are currently disease-specific and in context of the usual disease management process [83–85]. However, we found that certain conditions are prevalent across multiple HU groups, suggesting that the traditional disease-centred programs may be capturing a group of patients with the same diagnosis but with heterogeneity in utilization patterns and by extension, care needs. Such disease-centric programs may then be limited in effectiveness due to the inherent variation present in the patient populations treated and the hospital setting in which the disease is treated. For instance, diagnosis of acute cerebrovascular disease was found to be prevalent across the Cost, Cost-LOS and Cost-LOS-SOC HU groups. Care pathways have been commonly adopted for stroke management to improve patient care quality and outcomes [86]. While these programs may include outpatient treatment as part of the pathway, they generally focus on inpatient-related care during the acute phase. However, it is clear that there is a group of patients with cerebrovascular disease that have high outpatient needs, suggesting the need to look at a more holistic program that focuses not only on the inpatient aspect of stroke care, but extends to outpatient care as well for this group.

An effective program design should either accommodate the variation in the patient profiles, or target only a particular subgroup of patients. As patients at risk of high utilization often have high prevalence of multiple complex chronic conditions and not just one disease, new integrated models of care that are generic and disease agnostic, and that address the cross-cutting needs of a patient, may be more appropriate and effective in addressing high resource use across the different archetypes of HUs. Interventions such as case management, care planning and bundling of care have already been implemented in specific high-risk groups with complex needs such as older patients and patients with chronic diseases [87–92]. However, with increasing age, chronicity and complexity in the general population, applying this patient-centric approach across the different segments of the population will be better able to address the diverse health and social needs of each group [92, 93]. In parallel, the empirical approach in segmenting the patient population we have proposed would facilitate targeting of certain subgroups, by increasing within-group homogeneity in utilization profile and subsequently the relevance of any new interventions targeted at reducing high resource use.

Our findings highlight the importance of selecting the correct metrics in population segmentation. Selection can either be hypothesis-driven, with the intention to zoom in on a particular type of patient group, or pragmatically motivated by availability and access to information. Segmentation of patient populations is commonly achieved using clustering, but the segments have to be labelled post hoc given the characteristics of the identified clusters [39, 75, 76, 94, 95]. On the other hand, Cost, LOS and SOC data are convenient starting points for segmentation since they constitute the basic data collected for hospital databases and can be readily processed to generate intuitive and reproducible HU groups based on the 90th percentile of the cohort. While cost is a straightforward metric of resource use, broadening the definitions to include other metrics and further stratifying these HUs unveils the elevated resource use in other areas that would have been obscured. This representation of other non-high-cost HU groups highlight potential areas for improvement in current care processes which would have otherwise been missed, should the focus only be on high-cost groups.

Originating from one of the only two AMCs in Singapore, the data and analysis offers an important overview of HUs in an AMC in an Asian population. All patients above the age of 21 were included for analysis and information was collected at point of visit, minimising selection and information bias. Socio-demographic information was only available for the last visit in the system, and as changes to gender and ethnicity of the patients over the study period would have been minimal, only non-differential misclassification biases due to changes in housing type would be an inherent limitation in interpreting the information on SES of the patients. The reported healthcare costs in this study were estimated using patient bills as a proxy of cost, and do not reflect the true costs incurred by the hospital. However, as patient bills have been demonstrated to be positively correlated to costs across various studies [96–98], these billed charges are nonetheless a valid measure for the purpose of identifying high utilizers in our study. The use of observation years instead of calendar years allowed us to better account for resource use arising from disease progression over time. The associations seen between the first observed year HU groups and persistence of HU would only be generalizable to patients who survived the year. This study provides an extensive but incomplete comparison and description of HUs, as primary care data was not included, and we were not able to examine the implications of segmentation on primary care utilization. As the healthcare system in Singapore was reorganized in 2017, a group of polyclinics was integrated into the healthcare cluster and the inclusion of this primary care data for future work would complete the picture of HU groups in the cluster. Utilization of patients in other healthcare clusters was also not available, which would underestimate the total healthcare utilization accumulated by patients who seek care across multiple hospitals. A local study on three regional hospitals found that the rate of patients visiting all three hospitals was 8%, suggesting the need to take into account potential cross-utilization of patients in interpretation of our findings [99]. Generalizability of the characteristics of HUs to non-tertiary care settings would also be limited given that the study was based on an AMC. Taking into consideration the abovementioned limitations, this study nonetheless adds invaluable insight into the use of administrative data to segment a hospital-based patient population, and the profiles of patients with varying utilization patterns across the different hospital settings.

An extension of the segmentation approach illustrated in this study would be to segment a specific clinical subpopulation, examine the HU group distribution in this subpopulation, and compare these distributions across different clinical diagnoses. Further studies would also seek to expand on the persistence of HUs into subsequent years and distinguish the trajectories for each HU group. Effective identification and targeting of persistent users would maximise the use of resources channelled to these interventions, as patients who will revert to low resource use on their own over time will be omitted, and only patients who remain within the system and require the intervention will receive the program. These persistent users could be characterised and distinguished from the transient HUs, with the aim of informing program design to detect and target persistence in context of each group’s utilization patterns and disease profile. In addition, as we have examined the HU behaviour from the health system perspective in this study, a follow-up study examining patients with high out-of-pocket expenditure would be conducted to provide insight on high utilization from the patient’s perspective.

## Conclusion

High utilizers are a heterogeneous group of patients and there is a need to move beyond a one-size-fits-all metric to measure high utilization. We demonstrated the use of healthcare cost, as well as LOS and SOC utilization as metrics to identify different HU groups in a cohort of patients followed for 1 year. Differences in socio-demographic characteristics, multi-morbidity and disease profile were detected between the HU groups. Persistence of HU behavior in our study was pronounced in groups with high SOC utilization, and this trend was evident even after accounting for socio-demographic and clinical characteristics. These groups with high SOC utilization would be prime candidates for in-depth analysis of longitudinal behavior to distinguish persistent HUs from transient HUs, track their transitions to different HU groups in subsequent years, and determine groups feasible for intervention. Intervention design tackling excess resource use should take into consideration the inherent variation in utilization patterns among the patients and address the specific needs of each subgroup when developing an effective and targeted program. Segmentation of a patient cohort using these utilization metrics will enable policy makers to better identify the diverse needs of patients, detect gaps in current care and focus their efforts in delivering care relevant and tailored to each segment.

## Additional files

Additional file 1: Top 5 common conditions in Year 1 high utilizer (HU) groups by visit frequency. (DOCX 19 kb)

Additional file 2: Descriptive statistics for persistent and non-persistent high utilizers (HUs). (DOCX 19 kb)

## Data Availability

The datasets generated and/or analysed during the current study are not publicly available due to the Personal Data Protection Act enacted in Singapore, which prohibits the disclosure of identifiable data to the public. R programming codes used in the analysis are available from the corresponding author on reasonable request.

## Abbreviations

AMC: Academic Medical Center
CCMI: Charlson Comorbidity Index
CCS: Clinical Classification Software
CI: Confidence interval
ED: Emergency Department
EMR: Electronic medical record
HU: High utilizer
IQR: Interquartile range
LOS: Length of stay
LRT: Likelihood ratio test
OECD: Organisation for Economic Co-operation and Development
OR: Odds ratio
PPS: Polypharmacy score
SES: Socio-economic status
SOC: Specialist Outpatient Clinic
