# Supplementary material for: Characterization of high healthcare utilizer groups using administrative data from an electronic medical record database
Source: BMC Health Serv Res. 2019 Jul 5;19:452. doi: 10.1186/s12913-019-4239-2 (PMC6612067; doi:10.1186/s12913-019-4239-2)
Supplement: Supplementary file 1 — Top 5 common conditions in Year 1 high utilizer (HU) groups by visit frequency. (DOCX 19 kb) [file 12913_2019_4239_MOESM1_ESM.docx]

Top 5 common conditions in Year 1 high utilizer (HU) groups by visit frequency

| HU Group | Condition | Visits (% of Group) | Patients (% of Group) |
| --- | --- | --- | --- |
| Non-HU | Superficial injury; contusion | 28,928 (2.8%) | 27,049 (8.4%) |
| (1,051,001 visits | Other upper respiratory infections | 16,884 (1.6%) | 14,778 (4.6%) |
| 322,905 patients) | Sprains and strains | 15,737 (1.5%) | 14,546 (4.5%) |
|  | Open wounds of extremities | 14,089 (1.3%) | 11,447 (3.5%) |
|  | Nonspecific chest pain | 13,310 (1.3%) | 9,541 (3.0%) |
|  |  |  |  |
| Cost | Acute myocardial infarction | 4,591 (4.4%) | 2,877 (19.6%) |
| (104,518 visits, | Coronary atherosclerosis and other heart disease | 3,619 (3.5%) | 2,946 (20.1%) |
| 14,647 patients) | Essential hypertension | 3,515 (3.4%) | 781 (5.3%) |
|  | Disorders of lipid metabolism | 1,751 (1.7%) | 355 (2.4%) |
|  | Acute cerebrovascular disease | 1,608 (1.5%) | 879 (6.0%) |
|  |  |  |  |
| LOS | Mood disorders | 96 (8.8%) | 52 (24.0%) |
| (1086 visits, | Schizophrenia and other psychotic disorders | 65 (6.0%) | 36 (16.6%) |
| 217 patients) | Pneumonia^ | 21 (1.9%) | 13 (6.0%) |
|  | Residual codes; unclassified | 20 (1.8%) | 17 (7.8%) |
|  | Anxiety disorders | 19 (1.7%) | 11 (5.1%) |
|  |  |  |  |
| SOC | Other pregnancy and delivery including normal | 3,518 (0.9%) | 3,490 (13.3%) |
| (407,918 visits, | Essential hypertension | 2,876 (0.7%) | 548 (2.1%) |
| 26,179 patients) | Cataract | 1,923 (0.5%) | 1,379 (5.3%) |
|  | Fracture of upper limb | 1,628 (0.4%) | 1,055 (4.0%) |
|  | Diabetes mellitus without complication | 1,582 (0.4%) | 260 (1.0%) |
|  |  |  |  |
| LOS-SOC | Mood disorders | 89 (13.6%) | 23 (52.3%) |
| (653 visits, | Schizophrenia and other psychotic disorders | 35 (5.4%) | 14 (31.8%) |
| 44 patients) | Disorders of lipid metabolism | 16 (2.5%) | 1 (2.3%) |
|  | Other eye disorders | 7 (1.1%) | 5 (11.4%) |
|  | Adjustment disorders | 6 (0.9%) | 2 (4.5%) |
|  |  |  |  |
| Cost-LOS | Acute cerebrovascular disease | 1,857 (3.9%) | 1,049 (17.5%) |
| (47,279 visits, | Pneumonia^ | 1,550 (3.3%) | 838 (13.9%) |
| 6,008 patients) | Urinary tract infections | 996 (2.1%) | 546 (9.1%) |
|  | Congestive heart failure; nonhypertensive | 837 (1.8%) | 409 (6.8%) |
|  | Coronary atherosclerosis and other heart disease | 693 (1.5%) | 493 (8.2%) |
|  |  |  |  |
| Cost-SOC | Cancer of breast | 11,319 (3.2%) | 955 (7.4%) |
| (351,241 visits, | Female infertility | 5,415 (1.5%) | 650 (5.0%) |
| 12,892 patients) | Cancer of colon | 4,458 (1.3%) | 495 (3.8%) |
|  | Essential hypertension | 3,587 (1.0%) | 581 (4.5%) |
|  | Cancer of head and neck | 3,062 (0.9%) | 495 (3.8%) |
|  |  |  |  |
| Cost-LOS-SOC | Essential hypertension | 2,432 (1.4%) | 398 (7.6%) |
| (170,381 visits, | Non-Hodgkins lymphoma | 2,134 (1.3%) | 225 (4.3%) |
| 5,270 patients) | Chronic renal failure | 2,077 (1.2%) | 201 (3.8%) |
|  | Leukemias | 2,039 (1.2%) | 196 (3.7%) |
|  | Cancer of colon | 1,562 (0.9%) | 206 (3.9%) |

^ except that caused by tuberculosis or sexually transmitted disease
