# Supplementary material for: Characterization of high healthcare utilizer groups using administrative data from an electronic medical record database
Source: BMC Health Serv Res. 2019 Jul 5;19:452. doi: 10.1186/s12913-019-4239-2 (PMC6612067; doi:10.1186/s12913-019-4239-2)
Supplement: Supplementary file 2 — Descriptive statistics for persistent and non-persistent high utilizers (HUs). (DOCX 19 kb) [file 12913_2019_4239_MOESM2_ESM.docx]

Descriptive statistics for persistent and non-persistent high utilizers (HUs)

|  | Non-persistent HU (N=363,388) | Persistent HU (N=16,052) | P-value# |
| --- | --- | --- | --- |
| Year 1 HU group | |  |  |
| Non-HU | 312,057 (85.9%) | 6,223 (38.8%) | <0.001 |
| Cost | 12,507 (3.4%) | 680 (4.2%) |  |
| LOS | 177 (0.1%) | 20 (0.1%) |  |
| SOC | 22,421 (6.2%) | 3,668 (22.9%) |  |
| LOS-SOC | 33 (0.1%) | 11 (0.1%) |  |
| Cost-LOS | 3,818 (1.1%) | 564 (3.5%) |  |
| Cost-SOC | 9,543 (2.6%) | 3,102 (19.3%) |  |
| Cost-LOS-SOC | 2,832 (0.8%) | 1,784 (11.1%) |  |
|  |  |  |  |
| Age at first visit | 37 (27-51) | 51 (34-64) | <0.001 |
| CCMI | 0 (0-0) | 0 (0-2) | <0.001 |
| PPS | 2 (0-5) | 6 (2-15) | <0.001 |
|  |  |  |  |
| Gender |  |  | <0.001 |
| Female | 149,969 (41.3%) | 8,746 (54.5%) |  |
| Male | 213,419 (58.7%) | 7,306 (45.5%) |  |
|  |  |  |  |
| Race |  |  | <0.001 |
| Chinese | 210,299 (57.9%) | 10,415 (64.9%) |  |
| Indian | 48,096 (13.2%) | 1,807 (11.3%) |  |
| Malay | 42,256 (11.6%) | 1,954 (12.2%) |  |
| Others | 62,737 (17.3%) | 1,876 (11.7%) |  |
|  |  |  |  |
| Nationality | N=363,383 |  | <0.001 |
| Foreigner | 143,222 (39.4%) | 3,515 (21.9%) |  |
| Singaporean | 220,161 (60.6%) | 12,537 (78.1%) |  |
|  |  |  |  |
| Treatment type |  |  | <0.001 |
| Subsidised only | 246845 (67.9%) | 8,743 (54.5%) |  |
| Both subsidised and unsubsidised | 66,975 (18.4%) | 3,698 (23.0%) |  |
| Unsubsidised only | 49,568 (13.6%) | 3,611 (22.5%) |  |
|  |  |  |  |
| Housing type |  |  | <0.001 |
| 1/2-room flats | 9,080 (2.5%) | 538 (3.4%) |  |
| 3-room flats and larger | 227,387 (62.6%) | 11,677 (72.7%) |  |
| Private | 42,828 (11.8%) | 2,209 (13.8%) |  |
| Unknown | 84,093 (23.1%) | 1,628 (10.1%) |  |
|  |  |  |  |
| Common HU conditions | |  |  |
| Superficial injury contusion | 28,047 (7.7%) | 465 (2.9%) | <0.001 |
| Coronary atherosclerosis and other heart disease | 7,144 (2.0%) | 813 (5.1%) | <0.001 |
| Pneumonia^ | 3,485 (1.0%) | 414 (2.6%) | <0.001 |
| Non-infectious gastroenteritis | 12,787 (3.5%) | 389 (2.4%) | <0.001 |
| Sprains and strains | 15,390 (4.2%) | 337 (2.1%) | <0.001 |
| Fracture of lower limb | 6,191 (1.7%) | 209 (1.3%) | <0.001 |
| Intracranial injury | 7,312 (2.0%) | 294 (1.8%) | 0.117 |
| Fracture of upper limb | 9,850 (2.7%) | 203 (1.3%) | <0.001 |
| Cataract | 2,817 (0.8%) | 356 (2.2%) | <0.001 |
| Burns | 1,971 (0.5%) | 24 (0.1%) | <0.001 |
| Gastritis and duodenitis | 11,894 (3.3%) | 616 (3.8%) | <0.001 |
| Other upper respiratory infections | 15,450 (4.3%) | 477 (3.0%) | <0.001 |
| Urinary tract infections | 6,887 (1.9%) | 474 (3.0%) | <0.001 |
| Acute myocardial infarction | 3,965 (1.1%) | 576 (3.6%) | <0.001 |
| Residual codes unclassified | 3,916 (1.1%) | 355 (2.2%) | <0.001 |
| Normal pregnancy and delivery | 4,620 (1.3%) | 256 (1.6%) | <0.001 |
| Other eye disorders | 5,541 (1.5%) | 164 (1.0%) | <0.001 |
| Female infertility | 706 (0.2%) | 250 (1.6%) | <0.001 |
| Open wounds of extremities | 12,493 (3.4%) | 139 (0.9%) | <0.001 |
| Essential hypertension | 4,806 (1.3%) | 783 (4.9%) | <0.001 |
| Other complications of birth | 1,850 (0.5%) | 90 (0.6%) | 0.401 |
| Cancer of breast | 925 (0.3%) | 555 (3.5%) | <0.001 |
| Acute cerebrovascular disease | 4,352 (1.2%) | 567 (3.5%) | <0.001 |
| Septicemia (except in labor) | 1,086 (0.3%) | 262 (1.6%) | <0.001 |
| Mood disorders | 1,632 (0.4%) | 142 (0.9%) | <0.001 |
| Schizophrenia and other psychotic disorders | 611 (0.2%) | 63 (0.4%) | <0.001 |
| ***: p<0.001; ^ except that caused by tuberculosis or sexually transmitted disease; # Pearson’s Chi-square tests and Wilcoxon ranksum tests were used for categorical and continuous variables respectively | | | |
